# Increased blood levels of neutrophil- and platelet-derived markers in patients with radiographic axial spondyloarthritis: a pilot study

**DOI:** 10.1007/s00296-026-06090-8

**Published:** 2026-02-27

**Authors:** Neele K. Levin, Jonas Mårtensson, Lena Björkman, Per Venge, Eva Klingberg, Anna Deminger, Emma C. Josefsson, Huamei Forsman, Martina Sundqvist, Helena Forsblad-d’Elia

**Affiliations:** 1 Department of Rheumatology and Inflammation Research, Institute of Medicine, Sahlgrenska Academy, University of Gothenburg, Gothenburg, Sweden; 2 Department of Rheumatology, Sahlgrenska University Hospital, Region Västra Götaland, Gothenburg, Sweden; 3 Department of Medical Sciences, Uppsala University, Uppsala, Sweden; 4 Department of Clinical Chemistry, Sahlgrenska University Hospital, Region Västra Götaland, Gothenburg, Sweden; 5 Department of Laboratory Medicine, Institute of Biomedicine, Sahlgrenska Academy, University of Gothenburg, Gothenburg, Sweden

**Keywords:** Ankylosing spondylitis, Myeloperoxidase, CD40 ligand, P-selectin, Neutrophils, Blood platelets

## Abstract

A challenge in diagnosing and monitoring radiographic axial spondyloarthritis (r-axSpA) is the absence of reliable biomarkers. This cross-sectional pilot study aimed to evaluate platelet- and neutrophil-derived substances as potential biomarkers in r-axSpA, given the involvement of these cells in disease pathology. Serum and plasma were collected from 13 male, HLA-B27-positive r-axSpA patients without disease-modifying anti-rheumatic drugs and 13 age- and sex-matched blood donor controls. Concentrations of platelet-derived soluble CD40 ligand (sCD40L) and soluble P-selectin (sP-selectin) and neutrophil-derived human neutrophil lipocalin (HNL), myeloperoxidase (MPO), and galectin-3 were measured using ELISA. Associations between these markers and clinical parameters, including C-reactive protein (CRP) and erythrocyte sedimentation rate (ESR) were assessed by Spearman’s rank correlation. Group differences were analysed using the Wilcoxon signed-rank test. Median concentrations of serum sCD40L (4.2 vs. 2.7 ng/mL; *p* = 0.001), plasma HNL (23.7 vs. 18.6 mg/L; *p* = 0.040), serum HNL (74.7 vs. 52.3 mg/L; *p* = 0.013), and plasma MPO (56.6 vs. 37.3 mg/L; *p* = 0.027) were significantly higher in r-axSpA patients compared with controls. Plasma HNL correlated positively with CRP and ESR, but not with other clinical parameters. In both patients and controls, serum concentrations of sCD40L, sP-selectin, HNL, and MPO were significantly higher than their plasma counterparts, highlighting differences related to sample processing. Serum sCD40L, serum and plasma HNL, and plasma MPO were elevated in r-axSpA patients compared with controls. These findings warrant validation in larger cohorts to clarify the role of sCD40L, HNL and MPO across a heterogeneous group of patients with early and established r-axSpA.

**Supplementary Information:**

The online version contains supplementary material available at 10.1007/s00296-026-06090-8.

## Introduction

Radiographic axial spondyloarthritis (r-axSpA; also known as ankylosing spondylarthritis, AS) is a chronic immune-mediated rheumatic inflammatory disease, predominantly affecting the sacroiliac joints and spine. It is characterised by new bone formation, reduced mobility, impaired physical function, and extra-musculoskeletal manifestations [1, 2] and shows a strong genetic association with HLA-B27, which is present in 60–90% of patients [3]. A key challenge in diagnosis, prognosis and disease monitoring in r-axSpA is the lack of specific and reliable biomarkers, underscoring the need to identify novel markers.

Increased blood neutrophils have been reported in r-axSpA patients [4], and it is known that the soluble neutrophil granule-localised human neutrophil lipocalin (HNL; also known as neutrophil gelatinase-associated lipocalin [NGAL] or lipocalin 2) and myeloperoxidase (MPO) are considered indicators of systemic neutrophil activation [5, 6]. Platelets are likewise recognised as contributors to inflammation beyond haemostasis [7], with elevated platelet counts also observed in r-axSpA [4]. Among their α-granule proteins, soluble CD40 ligand (sCD40L) has been suggested as a pro-inflammatory biomarker in rheumatic disease [7, 8], while soluble P-selectin (sP-selectin) has been linked to inflammation and coronary artery disease [9]. However, their role in r-axSpA remains inconsistent [8, 10]. In addition, galectin-3, a β-galactoside-binding lectin expressed by neutrophils and multiple other cell types, has emerged as a pleiotropic pro-inflammatory mediator increasingly implicated in chronic inflammatory conditions, including r-axSpA [11, 12], suggesting a potential role in disease pathogenesis.

Based on these observations, we hypothesised that innate immune pathways are activated in r-axSpA, resulting in elevated blood levels of sCD40L, sP-selectin, HNL, MPO, and galectin-3. To test this, we conducted this cross-sectional pilot study comparing serum and plasma concentrations of these five proteins in male r-axSpA patients and age- and sex-matched blood donor controls. We also performed correlation analyses of these five protein levels with measures of disease activity and age.

## Materials and methods

This exploratory cross-sectional pilot study was conducted and reported in accordance with the STROBE guidelines of the EQUATOR Network. Thirteen male patients diagnosed with radiographic axial spondyloarthritis (r-axSpA) were selected at random from a larger, previously described cohort recruited from three rheumatology clinics in western Sweden [1, 2]. Inclusion criteria for the present study were ankylosing spondylitis according to the modified New York criteria [13], male sex, HLA-B27 positivity, and absence of disease-modifying anti-rheumatic drug (DMARD) treatment at the time of sampling. Exclusion criteria, predefined in the parent cohort, included psoriasis, inflammatory bowel disease, dementia, pregnancy, and difficulties in understanding Swedish [1, 2]. Thirteen age-and sex-matched blood donors served as controls. For serum samples, whole blood was collected in serum tubes without additive and for plasma samples, whole blood was drawn into tubes with ethylenediaminetetraacetic acid (EDTA) as anticoagulant. Both serum and plasma samples were centrifuged at 2300x*g* for 10 min, aliquoted and stored at – 20 °C within four hours post blood draw. For long-term storage samples were kept at – 80 °C. The human CD40L (soluble) ELISA kit (Invitrogen, catalogue #BMS293), and the human P-Selectin (soluble) (CD62) ELISA kit (Invitrogen, catalogue #BMS219-4) were purchased from Thermo Fisher Scientific through Life Technologies Europe BV (Stockholm, Sweden), and the assays were performed according to the manufacturer’s instructions. Galectin-3 levels were quantified using the Galectin-3 96-well Test Kit (BG Medicine, catalogue #12727) which was purchased from Corgenix (B-field, USA) and performed in accordance with the manufacturer’s guidelines. The concentrations of MPO and HNL were measured by ELISAs provided by Diagnostics Development Sweden AB (Uppsala, Sweden). The HNL-ELISA used measures all forms of HNL in plasma or serum i.e. monomeric, dimeric and heteromeric forms [14, 15].

All data analysis and statistical evaluations were conducted using GraphPad Prism (version 10.6.1, San Diego, CA, USA). Details of the specific statistical tests applied, and the definition of statistically significant differences, are delineated in the footnotes to the tables or the figure legend for each data set.

## Results

**Fig. 1:**
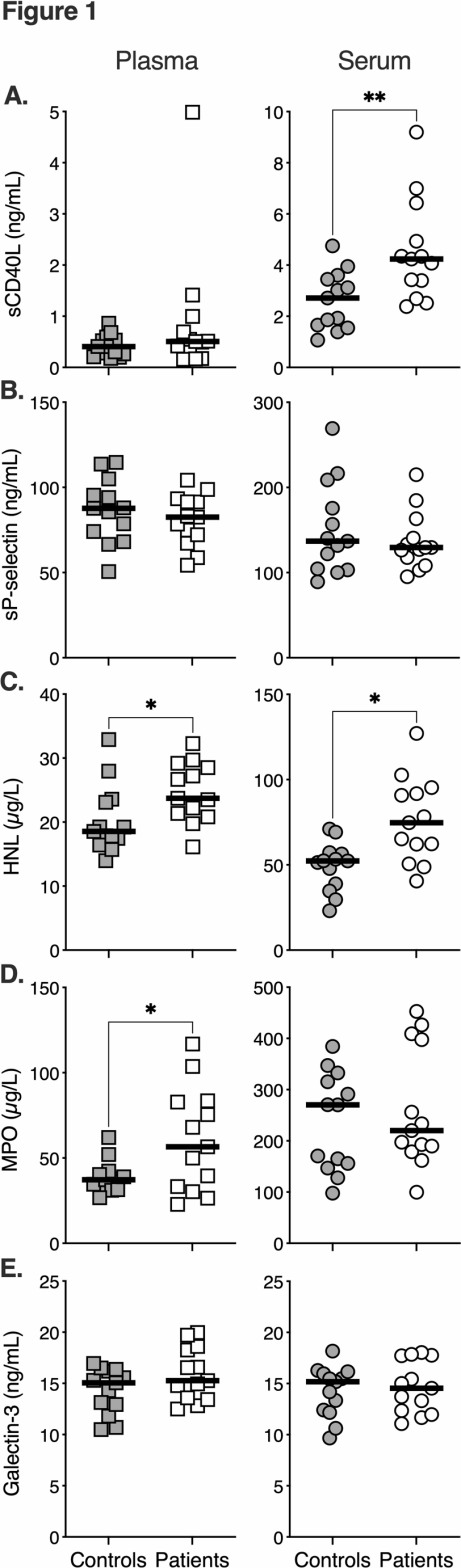
Blood levels of sCD40L, sP-selectin, HNL, MPO and galectin-3 in 13 male patients with r-axSpA compared to age-matched male controls. The concentrations of **A.** soluble CD40 Ligand (sCD40L), **B**. soluble P-selectin (sP-selectin), **C**. human neutrophil lipocalin (HNL), **D**. myeloperoxidase (MPO) or **E**. galectin-3 were measured in plasma (left panel) and serum (right panel). The data is presented as individual dots and a median (bold horizontal line). The levels of the analytes were compared between patients (white) and controls (grey) in plasma (squares) and serum (circles) samples separately. Statistical analysis was performed using the Wilcoxon signed-rank test, and statistically significant differences are denoted as *p < 0.05, **p < 0.01.

**Fig. 2:**
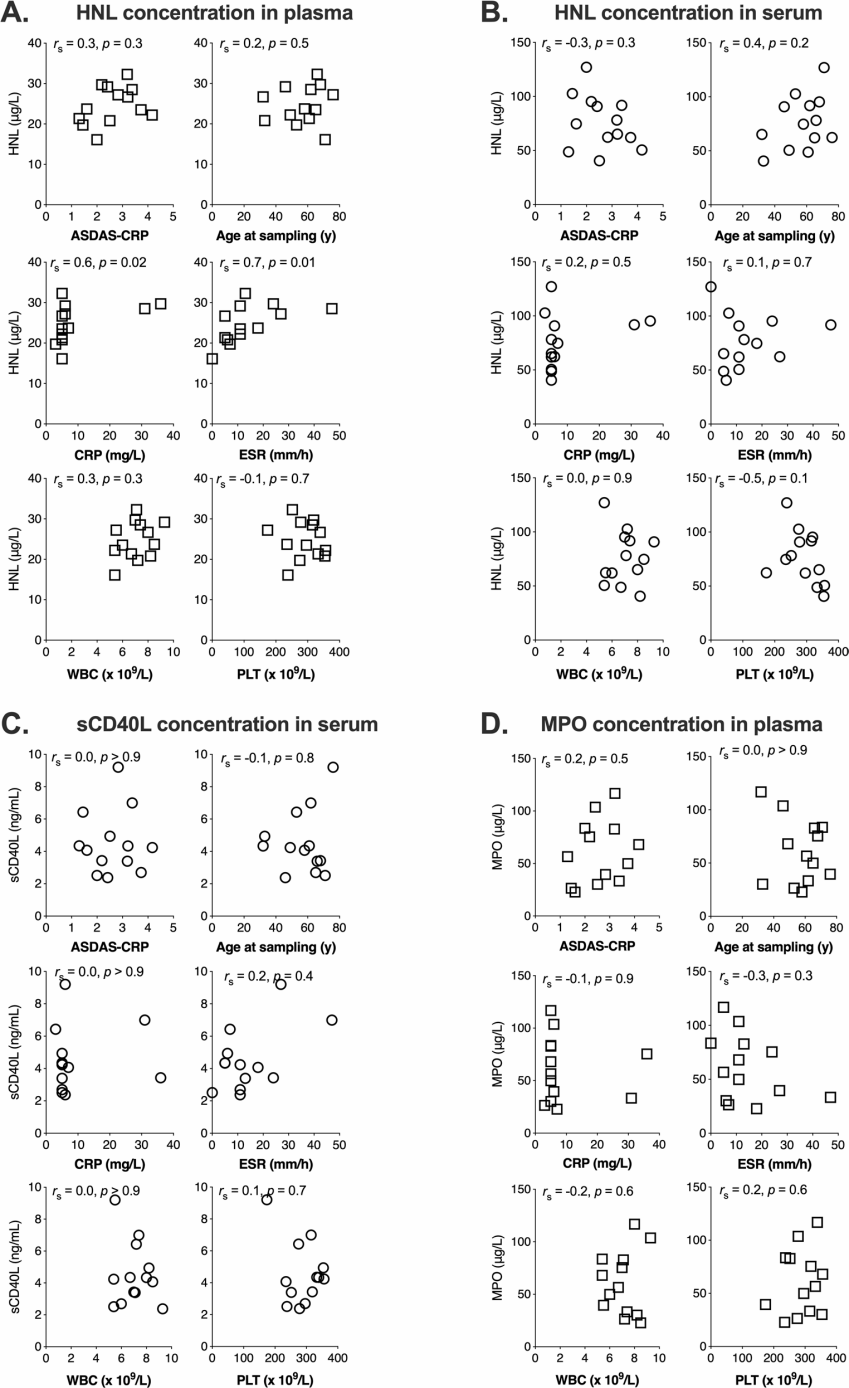
Scatter plots showing correlations between HNL, sCD40L or MPO levels and clinical characteristics in 13 male r-axSpA patients. **A–D**. Ankylosing Spondylitis Disease Activity Score with C-reactive protein (ASDAS-CRP), C-reactive protein (CRP), age at sampling, erythrocyte sedimentation rate (ESR), white blood cell count (WBC), and platelet count (PLT) were independently correlated with **A.** HNL plasma concentrations, **B**. HNL serum concentrations, **C**. sCD40L serum concentrations, and **D.** MPO plasma concentration. Statistical analysis was performed using Spearman’s rank correlation. The Spearman’s rank correlation coefficient (*r*_*s*_) and *p*-values (*p*) are stated in each scatter plot.

The primary results are summarised in Figs. 1 and 2; Table 2 and include group comparisons of platelet- and neutrophil-derived markers as well as correlation analyses with clinical and inflammatory parameters.

### Clinical characteristics

None of the included patients were treated with disease-modifying anti-rheumatic drugs (DMARDs), while 9 of 13 were on non-steroidal anti-inflammatory drugs (NSAIDs). Clinical characteristics of the patients and the age- and sex-matched blood donor controls are summarised in Table 1. Individual patient data are provided in online Supplementary Table S1.

**Table 1:** Clinical characteristics of r-axSpA patients and controls

Characteristics	Patients, *n* = 13	Controls, *n* = 13
Sex (male/female)	13/0	13/0
NSAID treatment (*n* (%))	9 (69)	n.a.
Age (years)	61 (48–67)^a^	61 (48–67)
Age at r-axSpA diagnosis (years)	32 (26–49)	n.a.
ASDAS-CRP (score)	2.5 (1.8–3.3)	n.a.
BASFI (score)	2.3 (1.2–5.3)	n.a.
BASMI (score)	3.0 (2.1–4.2)	n.a.
ESR (mm/h)	11.0 (6.0–21.0)	n.a.
CRP (mg/L)	5.0 (5.0–7.0)	n.a.
WBC (x 10^9^/L)	7.1 (5.8–8.1)	n.a.
PLT (x 10^9^/L)	296.0 (245.0–336.0)	n.a.

*ASDAS-CRP* Ankylosing Spondylitis Disease Activity Score with C-reactive protein, *BASFI* Bath Ankylosing Spondylitis Functional Index, *BASMI* Bath Ankylosing Spondylitis Metrology Index, *CRP* C-reactive protein, *ESR* erythrocyte sedimentation rate, *IQR* interquartile range, *n.a.* not applicable, *n* number, *NSAID* non-steroidal anti-inflammatory drug, *PLT* platelet count, *WBC* white blood cell count. Reference intervals for adult males: CRP < 3 mg/L; ESR < 50 years < 13 mm/h, ≥ 50 years < 20 mm/h; WBC 3.5–8.8 × 10^9^/L; PLT 145.0–348.0 × 10^9^/L.

^a^Data are expressed as median (IQR) values.

### Group comparisons of circulating markers

Median concentrations of serum sCD40L, plasma and serum HNL as well as plasma MPO were significantly higher in r-axSpA patients compared with controls. In contrast, plasma sCD40L, plasma and serum sP-selectin, serum MPO, and plasma and serum galectin-3 were similar between groups (Fig. 1; Table 2). White blood cell (WBC) and platelet (PLT) counts were within the normal range in r-axSpA patients (Table 1).

**Table 2:** Comparisons of neutrophil-and platelet-derived soluble factors in plasma and serum between patients with r-axSpA and controls

Soluble factor	Patients, *n* = 13			Controls, *n* = 13			Patients vs. controls	
	Plasma	Serum	*p*	Plasma	Serum	*p*	Plasma, *p*	Serum, *p*
sCD40L (ng/mL)	0.5 (0.2–0.9)	4.2 (3.0–5.7)	***	0.4 (0.2–0.6)	2.7 (1.6–35)	***	ns	**
sP-selectin (ng/mL)	82.6 (69.7–92.4)	129.6 (113.0–151.9)	***	87.7 (71.1–100.2)	137.0 (103.8–192.2)	***	ns	ns
HNL (µg/L)	23.7 (21.1–28.9)	74.7 (56.3–93.6)	***	18.6 (16.9–23.3)	52.3 (36.8–56.8)	***	*	*
MPO (µg/L)	56.6 (31.8–83.2)	220.0 (184.2–403.4)	***	37.3 (32.8–41.3)	270.1 (151.4–324.0)	***	*	ns
Galectin-3 (ng/mL)	15.3 (13.5–18.4)	14.6 (12.2–17.7)	**	15.1 (12.4–16.3)	15.2 (12.3–16.1)	ns	ns	ns

*sCD40L* soluble CD40 Ligand, *sP-selectin* soluble P-selectin, *HNL* human neutrophil lipocalin, *MPO* myeloperoxidase, *r-axSpA* radiographic axial spondyloarthritis. Data are expressed as median (interquartile range) values. Comparisons between groups were evaluated by Wilcoxon signed-rank test and are indicated as follows: *p < 0.05, **p < 0.01, ***p < 0.001, no statistically significant difference (ns) *p* ≥ 0.05.

Across both patients and controls, serum concentrations of sCD40L, sP-selectin, HNL, and MPO were significantly higher than corresponding plasma concentrations (Fig. 1; Table 2), consistent with differences between serum and plasma measurements [16, 17].

### Correlation analyses

Plasma HNL showed a significantly positive correlation with CRP (*r*_*s*_: 0.6, *p* = 0.02) and ESR (*r*_*s*_: 0.7, *p* = 0.01) but not with the other included measures. No significant correlations were observed between serum HNL, serum sCD40L or plasma MPO and age at sampling, ASDAS-CRP, CRP, ESR, WBC or PLT (Fig. 2).

## Discussion

This pilot study investigated neutrophil- and platelet-derived soluble markers as well as galectin-3 in patients with r-axSpA. We found significantly higher levels of HNL in both plasma and serum, as well as significantly elevated concentrations of plasma MPO and serum sCD40L, in r-axSpA patients compared with controls. In contrast, no differences were detected in neither plasma nor serum regarding sP-selectin and galectin-3. Across both studied groups, serum concentrations of granule-associated proteins exceeded those in plasma (except for galectin-3), consistent with clotting-induced release [16, 17].

Neutrophils are increasingly recognised as important players across the spondyloarthritis (SpA) spectrum [18]. We found that HNL, a sensitive marker of neutrophil degranulation [6], was significantly elevated in both plasma and serum, consistent with an earlier study demonstrating increased serum HNL in r-axSpA [19]. Our detection of increased plasma levels suggests circulating neutrophil activation in r-axSpA, with clotting-amplifying release in serum. Additionally, plasma MPO levels were also significantly elevated, consistent with previous findings of increased MPO (both mRNA and protein) in r-axSpA [5, 20]. Neutrophils release their granules in a hierarchical order, with the specific granules (containing HNL) being mobilised already under modest stimulation whilst the azurophilic granules (containing MPO) require a stronger activation for mobilisation to the plasma membrane [21]. Therefore, increased levels of both plasma HNL and MPO shows a sustained neutrophil activation in r-axSpA patients.

Previous research has linked platelet count and platelet-to-lymphocyte ratio to disease severity [4], and transcriptomic analyses have identified altered expression of immune-related genes, including CD40L [22]. In this study, serum sCD40L was significantly increased in r-axSpA patients despite normal platelet counts and treatment with NSAIDs, well-known inhibitors of platelet activation, supporting a pro-inflammatory role of CD40-CD40L signalling in autoimmunity [7]. However, a previous study reported conflicting results regarding sCD40L in r-axSpA [10]. In contrast, platelet-derived sP-selectin was not elevated, consistent with one earlier report [10] but in disagreement with another study linking increased sP-selectin mRNA expression to a poorer prognosis [23]. The reasons for this discrepancy remain speculative. As previously outlined, it may be influenced by the fact that 69% of patients in this pilot study received treatment with NSAIDs. It could also relate to the fact that mRNA expression is not invariably proportional to the amount of translated protein, or to differential regulation of platelet α-granule proteins. Mechanistically, both proteins are inserted into the plasma membrane post degranulation and then enzymatically cleaved (likely by different enzymes) from the platelet surface to produce soluble ectodomains [24] adding to the complexity when interpreting results. In the current study, serum sCD40L levels were elevated in r-axSpA indicating augmented CD40L expression or a pre-activated (primed) platelet phenotype, reinforcing the notion of platelet involvement in r-axSpA pathogenesis.

Galectin-3, produced by neutrophils and multiple other cell types including monocytes and epithelial cells, regulates inflammation and tissue remodelling [12]. Although elevated serum galectin-3 has previously been reported in r-axSpA [11], we observed no group differences. This may relate to methodological differences, as Cao et al. [11] used a chemiluminescent microparticle immunoassay rather than an ELISA. Interestingly, plasma galectin-3 levels were significantly, albeit modestly, higher than serum levels in patients with r-axSpA, whereas no such difference was observed in controls. This modest increase is not readily explained by clotting-induced release and may instead reflect enhanced plasma stability of galectin-3, although this remains speculative at present.

A strength of this study is the combined analysis of plasma and serum, allowing a more nuanced understanding of proteins released under anticoagulated versus clotting-induced conditions. The inclusion of multiple markers provided a broader perspective on cellular contributions to r-axSpA pathogenesis compared with studies restricted to single analytes. To minimise potential confounders, we focused on a homogeneous group of HLA-B27-positive male patients with established disease and without DMARD treatment. However, most patients were receiving NSAIDs at the time of sampling, which may influence platelet activation and therefore represents a potential confounding factor that should be considered when interpreting the platelet-related findings. Accordingly, this design and the small sample size limit generalisability, and the findings should therefore be interpreted with caution and viewed as exploratory and hypothesis-generating, warranting validation in larger, more heterogeneous r-axSpA populations. Moreover, as two of the included analytes are neutrophil-derived, the lack of data on absolute neutrophil counts represents a limitation that should be addressed in future studies. Nevertheless, HNL and MPO primarily reflect neutrophil activation rather than absolute neutrophil counts and may therefore provide complementary information on neutrophil involvement in r-axSpA. Despite the limited cohort, a positive correlation between plasma HNL and markers of systemic inflammation (CRP and ESR) was observed, suggesting that elevated plasma HNL may reflect inflammatory activity. None of the other elevated markers showed significant correlations with CRP, ESR, or other clinical parameters.

In conclusion, the results of this pilot study support the involvement of neutrophils and platelets in r-axSpA, with HNL, MPO, and sCD40L showing disease-associated increases. Future studies incorporating longitudinal sampling will be essential to evaluate the clinical utility of these markers for diagnosis and disease monitoring.

## Supplementary Information

Below is the link to the electronic supplementary material.

Supplementary Material 1

## Data Availability

The datasets generated and/or analysed during the current study are available from the corresponding author on reasonable request.

## Declarations

### Provenance and peer review

Not commissioned, externally peer reviewed.
