# Supplementary material for: Increased blood levels of neutrophil- and platelet-derived markers in patients with radiographic axial spondyloarthritis: a pilot study
Source: Rheumatol Int. 2026 Feb 27;46(3):55. doi: 10.1007/s00296-026-06090-8 (PMC12948798; doi:10.1007/s00296-026-06090-8)
Supplement: Supplementary file 1 — Supplementary Material 1 [file 296_2026_6090_MOESM1_ESM.pdf]

SUPPLEMENTARY INFORMATION

Supplementary Table S1. Individual characteristics of the 13 included HLA-B27-positive men with radiographic axial spondyloarthritis (r-axSpA)

Patient ID	1	2	3	4	5	6	7	8	9	10	11	12	13
**Age (years)**													
At inclusions	71	76	66	58	61	62	46	68	49	32	65	33	53
At diagnosis	59	50	26	56	37	25	32	40	25	31	30	16	47
**Scores**													
ASDAS-CRP	2.00	2.83	3.19	1.60	1.30	3.38	2.42	2.19	4.17	3.21	3.73	2.51	1.45
BASFI	5.38	6.65	1.48	0.78	1.00	2.30	1.36	0.00	6.94	3.19	5.30	4.27	2.00
BASMI	5.80	7.20	3.20	2.80	1.80	4.00	2.20	4.40	2.60	3.80	3.00	1.60	2.00
**Inflammatory markers**													
ESR (mm/h)	0	27	13	18	5	47	11	24	11	5	11	6	7
CRP (mg/L)	5	6	5	7	5	31	6	36	5	5	5	5	3
**Cell counts (x 10^9/L)**													
WBC	5.4	5.5	7.1	8.5	6.7	7.4	9.3	7.0	5.4	8.0	6.0	8.2	7.2
PLT	238	174	252	235	333	315	278	319	356	339	296	354	275
NSAIDs	†	†	–	–	†	§	–	§	§	¶	†,*	‡	–

ASDAS-CRP, Ankylosing Spondylitis Disease Activity Score with C-reactive protein; BASFI, Bath Ankylosing Spondylitis Functional Index; BASMI, Bath Ankylosing Spondylitis Metrology Index; ESR, erythrocyte sedimentation rate; CRP, C-reactive protein; WBC, white blood cell count; PLT, platelet count; NSAIDs, non-steroidal anti-inflammatory drugs; †, ketoprofen; §, diclofenac; ¶, etoricoxib; *, indometacin; ‡, naproxen; –, no NSAID treatment.

Reference intervals for adult males: CRP < 3 mg/L; ESR < 50 years < 13 mm/h, ≥ 50 years < 20 mm/h; WBC 3.5 – 8.8 x 10^9/L; PLT 145 – 348 x 10^9/L.
